# Local Hematocrit Fluctuation Induced by Malaria-Infected Red Blood Cells and Its Effect on Microflow

**DOI:** 10.1155/2018/8065252

**Published:** 2018-04-23

**Authors:** Tong Wang, Zhongwen Xing

**Affiliations:** ^1^Department of Mathematics, Nanjing University of Aeronautics and Astronautics, Nanjing 210016, China; ^2^School of Electronic Science and Engineering, Nanjing University, Nanjing 210093, China

## Abstract

We investigate numerically the microscale blood flow in which red blood cells (RBCs) are partially infected by* Plasmodium falciparum*, the malaria parasite. The infected RBCs are modeled as more rigid cells with less deformability than healthy ones. Our study illustrates that, in a 10 *μ*m microvessel in low-hematocrit conditions (18% and 27%), the* Plasmodium falciparum*-infected red blood cells (*Pf*-IRBCs) and healthy ones first form a train of cells. Because of the slow moving of the *Pf*-IRBCs, the local hematocrit (*H*_ct_) near the *Pf*-IRBCs is then increased, to approximately 40% or even higher values. This increase of the local hematocrit is temporary and is kept for a longer length of time because of the long RBC train formed in 27%-*H*_ct_ condition. Similar hematocrit elevation at the downstream region with 45%-*H*_ct_ in the same 10 *μ*m microvessel is also observed with the cells randomly located. In 20 *μ*m microvessels with 45%-*H*_ct_, the *Pf*-IRBCs slow down the velocity of the healthy red blood cells (HRBCs) around them and then locally elevate the volume fraction and result in the accumulation of the RBCs at the center of the vessels, thus leaving a thicker cell free layer (CFL) near the vessel wall than normal. Variation of wall shear stress (WSS) is caused by the fluctuation of local *H*_ct_ and the distance between the wall and the RBCs. Moreover, in high-hematocrit condition (45%), malaria-infected cells have a tendency to migrate to the edge of the aggregates which is due to the uninterrupted hydrodynamic interaction between the HRBCs and *Pf*-IRBC. Our results suggest that the existence of Pf-IRBCs is a nonnegligible factor for the fluctuation of hematocrit and WSS and also contributes to the increase of CFL of pathological blood flow in microvessels. The numerical approach presented has the potential to be utilized to RBC disorders and other hematologic diseases.

## 1. Introduction

Every year, millions of people worldwide are infected with malaria, a mosquito-borne infectious disease. It is estimated that the resulting deaths are 731,000 in 2015 alone. Although the rates of disease have decreased in recent years, there is still a long way to go before it is virtually eliminated. When infected by malaria,* Plasmodium falciparum*, the parasite, invades and develops inside the human red blood cells (RBCs) and makes them stiffer than heathy cells [1–3]. Experimental study in which the deformability was assessed by measuring the pressure and time required for cell entry into 3 *μ*m pipettes has shown that the deformability decreases approximately 20-fold as rings mature to the schizont stage [4]. This change of deformability of healthy red blood cells (HRBCs) is due to the alternation of the cell membrane and cytoskeleton by the parasite [5, 6]. The modified blood rheology, raised flow resistance, and vasoocclusion may be the direct effect of this change in biomechanical property of infected RBCs [7, 8]. Thus, a realistic model for malaria-infected blood flow has to incorporate cell membrane stiffening [3, 9–11].

Under physiological conditions, HRBCs undergo lateral migration and thus form a dense red blood cell region near the center and create a cell free layer (CFL) close to the blood vessel wall [12]. When* Plasmodium falciparum*-infected red blood cells (*Pf*-IRBCs) are presented together with HRBCs, they are apt to move away from the vessel center and this phenomenon is called margination. Margination effect was first reported for leukocytes in blood flow [13]. This interesting aspect of the parasite-infected RBCs in malaria has been investigated experimentally [14, 15] and numerically [16]. Moreover, the *Pf*-IRBCs may adhere to microvasculature in postcapillary venules and arterioles [14, 17].

Microfluidic devices provide promising opportunities for the monitoring of the behavior of *Pf*-IRBCs in microflow [8, 18, 19]. In [18], a microfluidic apparatus with a row of triangular barriers was fabricated and the traversal of *Pf*-IRBCs was considered by the experimental and simulation study in fluid flow. It was observed that *Pf*-IRBCs traveled on average slower than HRBCs due to raised viscosity and membrane stiffness. In addition, microfluidic devices have been utilized in manipulating RBCs in other blood diseases, such as sickle-cell disease [20]. Although it is difficult to catch the cell-cell interactions using current experimental methods due to the complexity of the cell membrane, directly observing and quantifying the interactions of blood cells in microchannels have been conducted by* in vitro* investigations [21–24]. In these studies, effect of hematocrit and geometry [21] as well as temperature [24] on RBC radial dispersion in microchannels has been studied and quantified. Interaction between two RBCs [22] and the iteration of fluid particles with RBCs can also be identified [23].

One of the advantages of numerical models is their predictive capability and they can be applied to a number of diseases related to blood flow (such as malaria [3, 9, 10, 25, 26] and sickle-cell disease [27–30]). Numerical study [16] explained the mechanism of the margination effect of *Pf*-IRBCs and predicted that it might enhance cytoadhesion. Factors that affect the margination process have also been studied numerically [31]. Numerical methods have also been used to quantitatively predict the flow resistance through relative apparent viscosity in microvessels [9].

In the present study, a numerical model of microscale blood flow with malaria infection has been developed. We investigated the hydrodynamic interaction between *Pf*-IRBC and HRBCs in two-dimensional channels under different hematocrit (*H*_ct_) conditions. We adopted two-dimensional scheme because we believe that two-dimensional simulations are still helpful as a tool for preliminary research to aid conceptual understanding of microscale blood flow, especially for large systems where three-dimensional simulations are extremely costly. It can qualitatively capture the behavior of RBCs and may provide a guidance for future research. The numerical technique is in general able to be employed to those diseases and abnormalities where mechanical characteristics of cells have been altered.

## 2. Mathematical Models and Numerical Methods

The particulars of the red blood cell model in the present study were previously reported in [32, 33], and only a short interpretation is offered here. Presuming that cytoplasm of the red blood cells and blood plasma are Newtonian and incompressible fluids. The equations governing the blood flow in a rectangle domain *Ω*_*f*_ are given by

**Figure 1:**
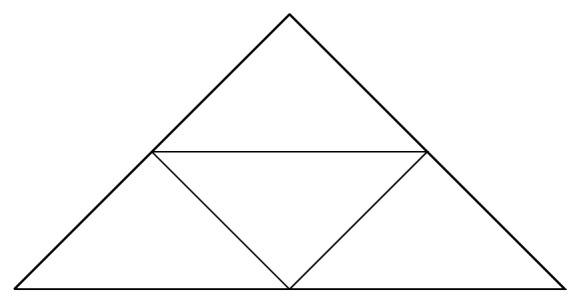
Schematic representation of the triangular mesh used in the simulations.

(1) ρ∂u∂t+u·∇u=−∇p+μΔu+f,in  Ωf,

(2) ∇·u=0,in  Ωf,

where the notation *t* is for time, *ρ* for density, **u** for velocity, *p* for pressure, *μ* for dynamic viscosity, and **f** for external force. No-slip boundary condition is enforced at the channel walls. In the direction of flow, periodic boundary condition is applied. The solution method of (1) and (2) has been thoroughly described elsewhere [34], where a uniform finite element triangulation (Figure 1) has been utilized. A RBC's membrane is simulated using a spring model. In the instance of a *Pf*-IRBC, a stiff cell is represented through increasing the membrane constants of the RBC model. This model was confirmed in the previous study to well simulate the deformation of RBCs in shear and Poiseuille flow [32]. The coupling of the deformable RBCs with fluid flow is achieved utilizing the immersed boundary method (IBM). The spring model for the RBCs and the immersed boundary method are reviewed briefly for completeness.

### 2.1. Red Blood Cell Model

The RBC membrane is simulated by *N* vertices that are joined through springs, where each vertex is depicted using a membrane particle. The model takes into consideration the elasticity and bending modulus, as well as constraints of enclosed area as follows:

(3) $$\begin{array}{c} E=E_{l}+E_{b}+\Gamma_{s}, \end{array}$$

where

(4) $$\begin{array}{c} E_{l}=\frac{k_{l}}{2}\overset{N}{\underset{i=1}{\sum }}{\left(\;\frac{l_{i}-l_{0}}{l_{0}}\;\right)}^{2} \end{array}$$

is the elastic energy and the bending energy is modeled by

(5) $$\begin{array}{c} E_{b}=\frac{k_{b}}{2}\overset{N}{\underset{i=1}{\sum }}\tan^{2}\left(\;\frac{\theta_{i}}{2}\;\right). \end{array}$$

In (4) and (5), *N* denotes number of springs; *k*_*l*_ and *k*_*b*_ denote spring moduli due to changes in length and bending angle between two adjacent springs, respectively; *l*_*i*_ is the time-dependent length of the *i*th spring element and *l*_0_ is its reference length; and *θ*_*i*_ is the angle that is formed by two adjoined springs at the *i*th particle. In addition, by taking into consideration the area conservation constraint, the penalty function is given as [35]

(6) $$\begin{array}{c} \Gamma_{s}=\frac{k_{s}}{2}{\left(\;\frac{s-s_{e}}{s_{e}}\;\right)}^{2}, \end{array}$$

with *s*_*e*_ and *s* being the equilibrium area and the time-dependent area of the RBC, respectively. Details of applying this spring model to obtain the shape of the RBCs can be found elsewhere [32, 33].

### 2.2. The Immersed Boundary Method

The cell-plasma interaction was coped with by the immersed boundary method (IBM) [36], in which the deformable object's boundary is estimated as follows: firstly, the force term at the membrane node **X** = {*X*_1_, *X*_2_} influences the nearby fluid nodes denoted by **x** = {*x*_1_, *x*_2_} through a discrete delta function *δ*:

(7) $$\begin{array}{c} F\left(\;x\;\right)=\underset{x}{\sum }F\left(\;X\;\right)D_{h}\left(\;X-x\;\right),\;\text{for }\left|X-x\right|\leq 2h, \end{array}$$

where *h* denotes the mesh size and

(8) $$\begin{array}{c} D_{h}\left(\;X-x\;\right)=\delta_{h}\left(\;X_{1}-x_{1}\;\right)\delta_{h}\left(\;X_{2}-x_{2}\;\right). \end{array}$$

The discrete delta function *δ* is

(9) $$\begin{array}{c} \delta_{h}\left(\;z\;\right)=\left\{\begin{array}{cc} \frac{1}{4h}\left(1+\cos\left(\frac{\pi z}{2h}\right)\right) & \text{for }\left|z\right|\leq 2h,\\ 0 & \text{for }\left|z\right|>2h. \end{array}\right. \end{array}$$

The force in (7) is coupled in (1); next, the membrane velocity is influenced by the adjacent fluid field using the same delta function:

(10) $$\begin{array}{c} U\left(\;X\;\right)=\sum h^{2}u\left(\;x\;\right)\cdot D_{h}\left(\;X-x\;\right)\;\text{for }\left|X-x\right|\leq 2h. \end{array}$$

Lastly, the location of the membrane is renewed by

(11) $$\begin{array}{c} X_{t+\Delta t}=X_{t}+△tU\left(\;X_{t}\;\right). \end{array}$$

## 3. Problem Description

In this paper, flows were simulated in a rectangular channel with a height of 10 *μ*m and 20 *μ*m in *y*-direction. The horizontal width of the channel was specified at 30 *μ*m for the simulations, but the periodic boundary condition was engaged to detect RBCs' long-term behavior in flow. The flow was pushed by a pressure gradient between the inlet and outlet. In the computational domain (10 *μ*m × 30 *μ*m), we included a *Pf*-IRBC and some HRBCs. The healthy RBCs had membrane constants *k*_*b*_ = *k*_*l*_ = 1.0 × 10^−12^ Nm, and the *Pf*-IRBC was characterized as 20-fold membrane stiffness [4]. To examine the influences of *H*_ct_ on the *Pf*-IRBC, we studied the flow for three different hematocrit values in this 10 *μ*m channel, *H*_ct_ = 18%, *H*_ct_ = 27%, and *H*_ct_ = 45%, and also a single *Pf*-IRBC without HRBCs. The number ratio of the *Pf*-IRBC to HRBCs for the three cases was then 1 : 3, 1 : 5, and 1 : 9, respectively. In the computational domain (20 *μ*m × 30 *μ*m), we included several *Pf*-IRBCs and some HRBCs. We compared the flows of two cases in the 20 *μ*m channel: *H*_ct_ = 45%, and one *Pf*-IRBC; *H*_ct_ = 45%, and two *Pf*-IRBCs. The number ratio of the *Pf*-IRBC to HRBCs was then 1 : 19 and 1 : 9, respectively. For clarity, the parameters that are adopted in the present investigation are listed in Table 1.

## 4. Results

### 4.1. Mesh Independence Study

**Figure 2:**
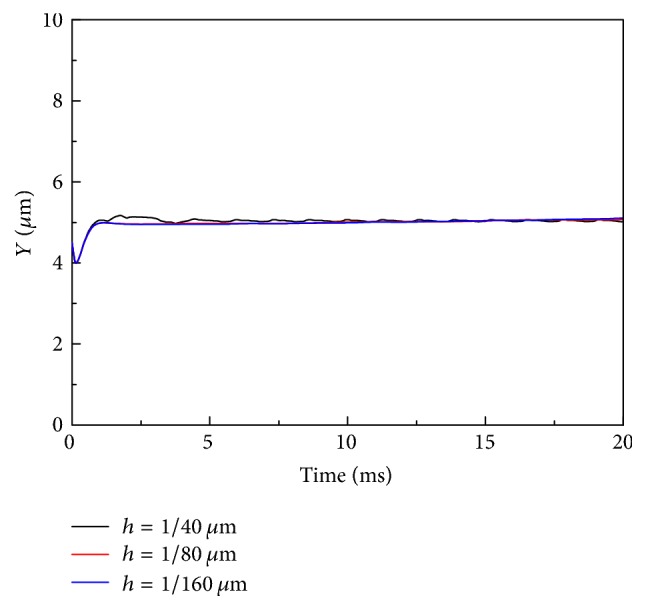
History of the cell center of a HRBC in the 10 *μ*m channel at different mesh size *h* values.

A mesh independence study has been conducted in the 10 *μ*m by 30 *μ*m channel by varying the mesh size *h* with a single HRBC and the results are shown in Figure 2. Three values of mesh size, that is, *h* = 1/40 *μ*m, *h* = 1/80 *μ*m, and *h* = 1/160 *μ*m, were chosen and the trajectories of the cell center of the HRBC are plotted versus time. The other parameters were kept unchanged and the values used are presented in Table 1. The analysis shows that the HRBCs migrate to the centerline of the channel in the flow when they are located off the centerline initially and this result is independent of the mesh size employed in the simulations. Therefore a mesh size of *h* = 1/80 *μ*m has been selected for all the simulations in this study.

### 4.2. Variation of Hematocrit and Wall Shear Stress (WSS)

**Figure 3:**
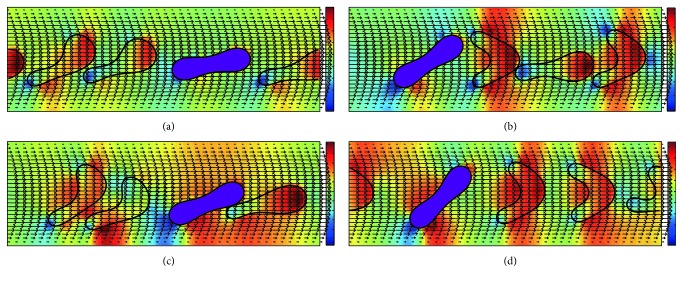
Successive snapshots of malaria-infected RBCs with *H*_ct_ = 18% in the 10 *μ*m channel. The purple cell represents the *Pf*-IRBC. The number ratio of *Pf*-IRBC to HRBC is 1 : 3. The time instants for simulation are (a) *t* = 5 ms, (b) *t* = 10 ms, (c) *t* = 20 ms, and (d) *t* = 25 ms. The pressure field is also illustrated.

Figure 3 displays the motion and blood flow behaviors in the 10 *μ*m channel with *H*_ct_ = 18% and the number ratio of *Pf*-IRBC to HRBC 1 : 3. Note the value of *H*_ct_ is significantly lower and the number ratio of *Pf*-IRBC to HRBC is much higher than in a patient. The purpose of including this configuration is to remove the hematocrit effect but to study the hydrodynamic effect of a *Pf*-IRBC on other HRBCs. The *Pf*-IRBC is presented by the purple cell. The pressure field is also shown in the same plot. Because *H*_ct_ is relatively low, the interaction between the HRBCs and *Pf*-IRBC is weak. The RBCs form a loose “train” formation so that the cell distribution is almost uniform. The *Pf*-IRBC deforms less than HRBCs and maintains its original biconcave shape while the HRBCs deform into various shapes, such as a parachute, slipper, or bullet. The *Pf*-IRBC and neighbor HRBC can touch and immediately separate; thus the interaction is not continuous.

**Figure 4:**
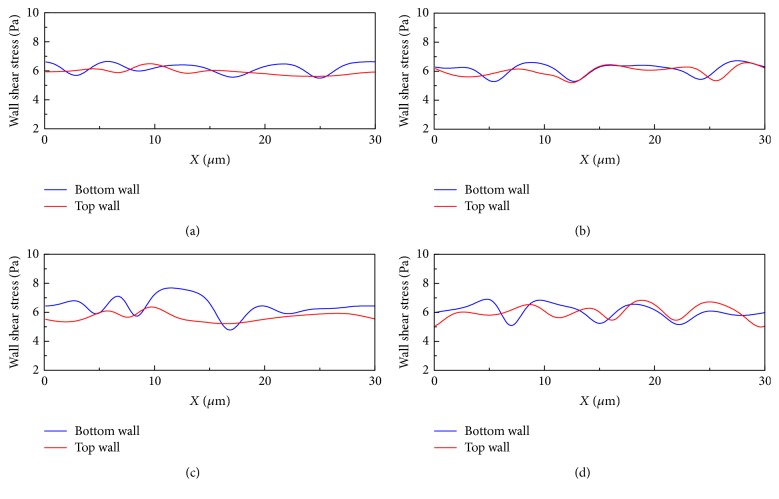
Wall shear stress variation along the channel walls for the simulation of malaria-infected RBCs with *H*_ct_ = 18% in the 10 *μ*m channel. The number ratio of *Pf*-IRBC to HRBC is 1 : 3. The time instants for simulation are (a) *t* = 5 ms, (b) *t* = 10 ms, (c) *t* = 20 ms, and (d) *t* = 25 ms (the time instants correspond to the same time instants in Figure 3).

It can also be observed from Figure 3 that the flow field around the cells is disturbed and this disturbance will introduce corresponding change in shear stress on the channel walls.  Figure 4 demonstrates the wall shear stress (WSS) variation along the channel walls for the same simulation condition in Figure 3. An interesting peak-valley-peak structure of WSS has been observed. The valley regions on the WSS curve correspond to the RBC that is close to the vessel wall, while the peak regions correspond to the cell-cell gap.

**Figure 5:**
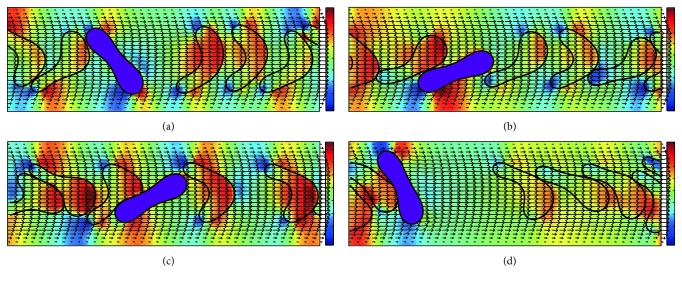
Successive snapshots of malaria-infected RBCs with *H*_ct_ = 27% in the 10 *μ*m channel. The purple cell represents the *Pf*-IRBC. The number ratio of *Pf*-IRBC to HRBC is 1 : 5. The time instants for simulation are (a) *t* = 5 ms, (b) *t* = 10 ms, (c) *t* = 20 ms, and (d) *t* = 50 ms. The pressure field is also illustrated.

**Figure 6:**
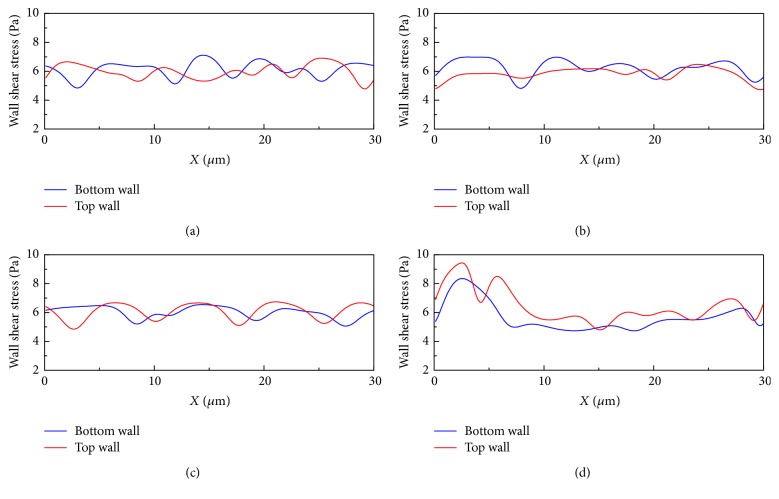
Wall shear stress variation along the channel walls for the simulation of malaria-infected RBCs with *H*_ct_ = 27% in the 10 *μ*m channel. The number ratio of *Pf*-IRBC to HRBC is 1 : 5. The time instants for simulation are (a) *t* = 5 ms, (b) *t* = 10 ms, (c) *t* = 20 ms, and (d) *t* = 25 ms (the time instants correspond to the same time instants in Figure 5).

When *H*_ct_ is 27% as shown in Figure 5, a more compact train is observed and thus the *H*_ct_ value at the train region can temporally increase to a higher value. It can be noticed from Figure 6 that the cell-wall gap size is the major factor influencing the WSS variation magnitude. Due to the narrow gap between the channel wall and cell membrane shown in Figure 5(d), the WSS variation in Figure 6(d) is most profound among all subfigures.

**Figure 7:**
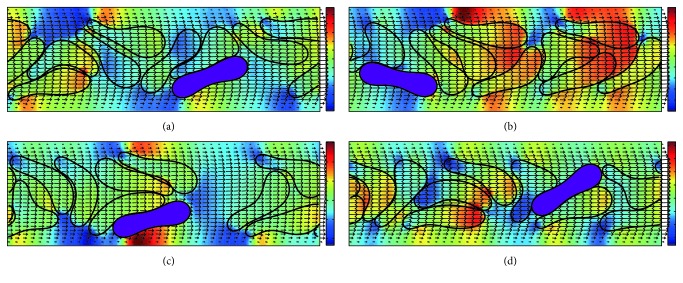
Successive snapshots of malaria-infected RBCs with *H*_ct_ = 45% in the 10 *μ*m channel. The purple cell represents the *Pf*-IRBC. The number ratio of *Pf*-IRBC to HRBC is 1 : 9. The time instants for simulation are (a) *t* = 5 ms, (b) *t* = 10 ms, (c) *t* = 15 ms, and (d) *t* = 20 ms. The pressure field is also illustrated.

**Figure 8:**
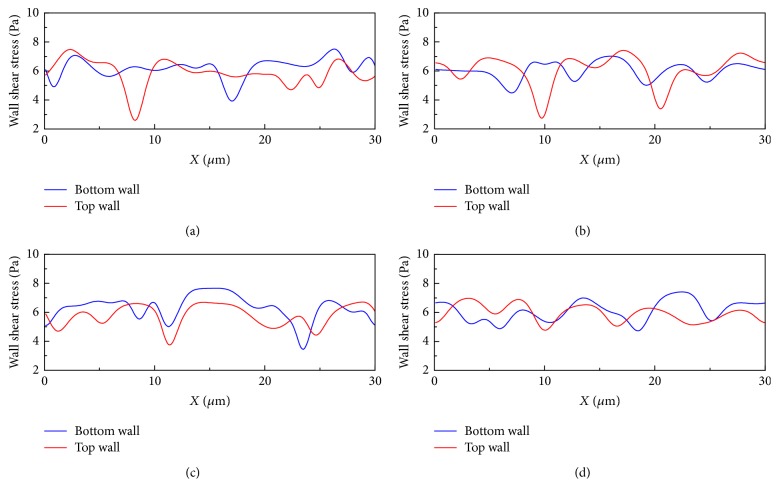
Wall shear stress variation along the channel walls for the simulation of malaria-infected RBCs with *H*_ct_ = 45% in the 10 *μ*m channel. The number ratio of *Pf*-IRBC to HRBC is 1 : 9. The time instants for simulation are (a) *t* = 5 ms, (b) *t* = 10 ms, (c) *t* = 20 ms, and (d) *t* = 25 ms (the time instants correspond to the same time instants in Figure 7).

In contrast, the flow behavior of cells in *H*_ct_ = 45% is different (Figure 7). We also observe *H*_ct_ elevation at the downstream region in this high-*H*_ct_ condition. The cells do not form a train but squeeze together with the cells being randomly located. This is for the reason that the HRBCs are frequently moving faster than the single *Pf*-IRBC and they are pushed by the following HBRCs to pass the *Pf*-IRBC in the gap between the cells and the wall. When this happens, the *Pf*-IRBC is forced to locate and flow near the vessel wall. The local *H*_ct_, which is defined as the hematocrit in a 10 *μ*m length of the channel centered at the *Pf*-IRBC, can increase as well (Figure 7(c)), but however may not last very long. The corresponding WSS shown in Figure 8 for this case varies much more severely compared to the low-*H*_ct_ cases.

**Figure 9:**
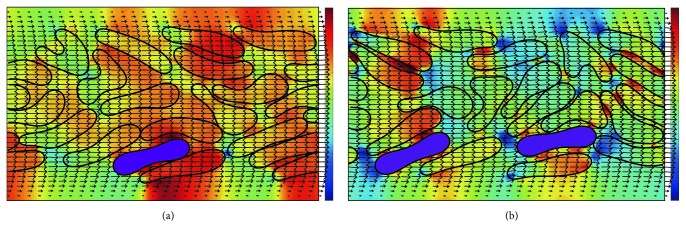
Typical configurations of RBCs with *H*_ct_ = 45% in the 20 *μ*m channel: (a) time instant *t* = 20 ms; the number ratio of *Pf*-IRBC to HRBC is 1 : 19; (b) time instant *t* = 40 ms; the number ratio of *Pf*-IRBC to HRBC is 1 : 9. The purple cells represent the *Pf*-IRBCs. The pressure field is also illustrated.

**Figure 10:**
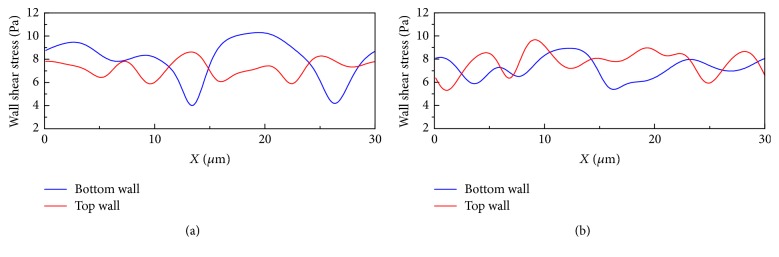
Wall shear stress variation along the channel walls for the simulation of malaria-infected RBCs with *H*_ct_ = 45% in the 20 *μ*m channel: (a) at time instant *t* = 20 ms; the number ratio of *Pf*-IRBC to HRBC is 1 : 19; (b) at time instant *t* = 40 ms; the number ratio of *Pf*-IRBC to HRBC is 1 : 9 (the time instants correspond to the same time instants in Figure 9).

Then we simulated blood flow with *H*_ct_ = 45% and the number ratio of 1 : 19 and 1 : 9 in the 20 *μ*m channel, and the results are presented in Figure 9 which illustrates typical cell distribution in this geometry. The HRBCs deform more than the *Pf*-IRBCs. The *Pf*-IRBCs move slower but however do not cause significant *H*_ct_ fluctuation. It is also noted that the pressure field changes with the motion of the RBCs. The cell depletion region causes a low pressure zone, while the pressure is relatively higher for the cell accumulating region. This pressure difference is more obvious in Figures 5(d), 7(c), and 9(a). It is also worth noting that the WSS in the 20 *μ*m channel experiences more profound variation (Figure 10).

**Figure 11:**
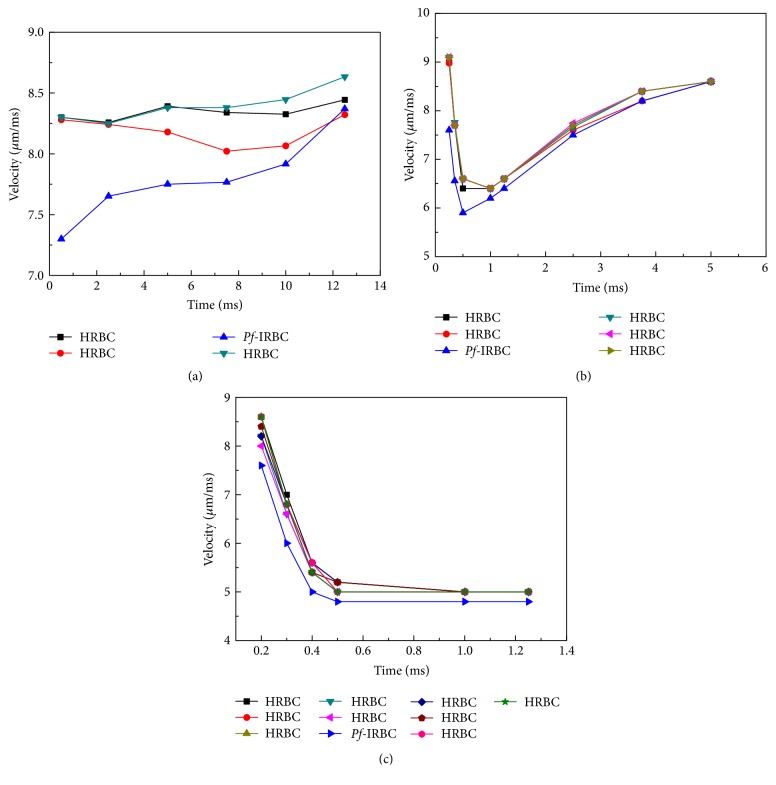
Instantaneous velocity of *Pf*-IRBC and HRBCs for a period of time in the 10 *μ*m channel with (a) *H*_ct_ = 18%, (b) *H*_ct_ = 27%, and (c) *H*_ct_ = 45%.

Figure 11 shows instantaneous velocity of Pf-IRBC compared to those of HRBCs for a period of time in the 10 *μ*m channel. In the blood with 18%-*H*_ct_, the *Pf*-IRBCs move much slower than HRBCs at the beginning of the simulation. Thus the HRBCs downstream and next to the *Pf*-IRBC (represented by the red line in Figure 11(a)) caught up with the *Pf*-IRBC and pushed it forward. The velocity of the *Pf*-IRBC then increases to almost the same as the downstream HRBC. Note that, in the 27%-*H*_ct_ blood, all the HRBCs are affected by the *Pf*-IRBC and slow down at the beginning of the simulation. All the cells move at about the same velocity when the velocities of the infected cell and noninfected cells reach the same value. The situation in the 45%-*H*_ct_ blood is quite different. Because of the compact configuration of the cells in the channel, although the velocity of the *Pf*-IRBC is smaller, the difference is not dramatic. The results further confirm that the *Pf*-IRBCs move slower than HRBCs in microscale blood flow and the *H*_ct_ level plays an important role in it.

**Figure 12:**
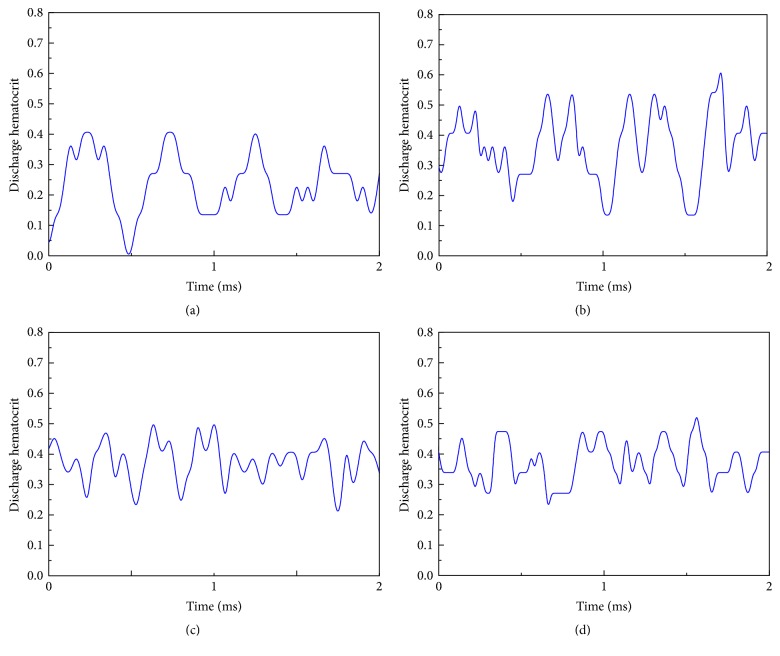
Variation of the discharge hematocrit for an arbitrary time period of 2 ms for the following: (a) *H*_ct_ = 27% in the 10 *μ*m channel; the number ratio of *Pf*-IRBC to HRBC is 1 : 5; (b) *H*_ct_ = 45% in the 10 *μ*m channel; the number ratio of *Pf*-IRBC to HRBC is 1 : 9; (c) *H*_ct_ = 45% in the 20 *μ*m channel; the number ratio of *Pf*-IRBC to HRBC is 1 : 19; (d) *H*_ct_ = 45% in the 20 *μ*m channel; the number ratio of *Pf*-IRBC to HRBC is 1 : 9.

To further quantify the effect of *Pf*-IRBCs on the variation of hematocrit, we examined the discharge hematocrit at the channel outlet. Discharge hematocrit *H*_*d*_ is the volume fraction of RBCs measured at the vessel exit and is defined in our simulations as follows:

(12) $$\begin{array}{c} H_{d}=\frac{{\bar{v}}_{c}}{\bar{v}}H_{\text{ct}}, \end{array}$$

where ${\bar{v}}_{c}$ and $\bar{v}$ denote the average cell speed and average flow speed, respectively.  Figure 12 compares the instant *H*_*d*_ in simulations for a time period of 2 ms with different *H*_ct_ values in the two microvessels. In general, the variation of *H*_*d*_ is more dramatic in the 10 *μ*m channel than in the 20 *μ*m channel. In the 10 *μ*m channel for the 27%-*H*_ct_ case, the discharge hematocrit is locally raised, to roughly 40% in the upstream zone of the *Pf*-IRBC. Such a high volume fraction of RBCs is caused by the turbulent flow owing to the slowly moving *Pf*-IRBC; thus, the discharge hematocrit turns substantially lower downstream, and it can sometimes be as low as ~0% in the cell void region. Similar variation of *H*_*d*_ for the 45%-*H*_ct_ blood in the same channel is observed. However, because of the relatively high value of *H*_ct_, the highest and the lowest *H*_*d*_ recorded for the time period are ~60% and ~13%, respectively. On the other hand, in the 20 *μ*m channel with 45%-*H*_ct_, the highest and the lowest *H*_*d*_ recorded for the time period are ~50% and ~22%, respectively. The number ratio of *Pf*-IRBC to HRBC seems to have no effect on the discharge hematocrit, although it does have an effect on the flow behavior.

### 4.3. Cell Free Layer (CFL) and Margination of *Pf*-IRBC

**Figure 13:**
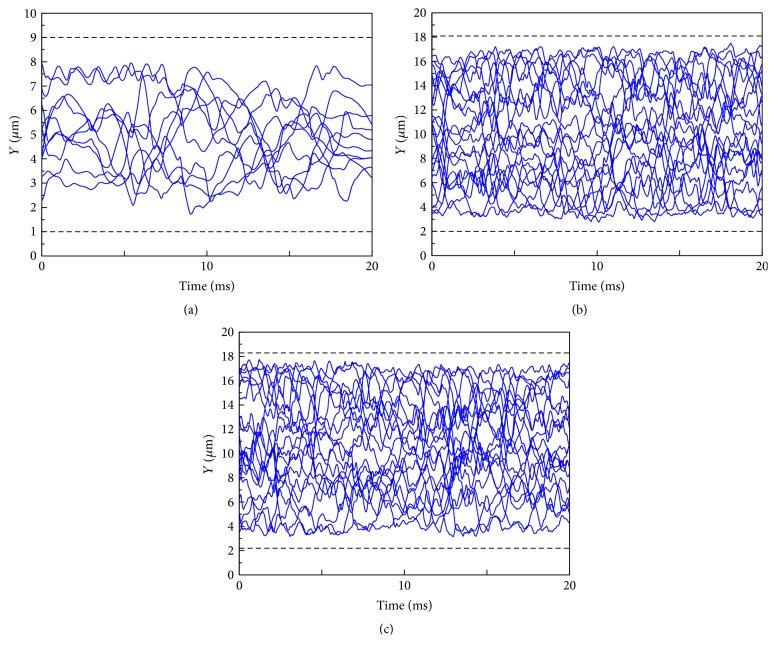
Trajectories of cell center for an arbitrary time period of 20 ms for the following: (a) *H*_ct_ = 45% in the 10 *μ*m channel; the number ratio of *Pf*-IRBC to HRBC is 1 : 9; (b) *H*_ct_ = 45% in the 20 *μ*m channel; the number ratio of *Pf*-IRBC to HRBC is 1 : 19; (c) *H*_ct_ = 45% in the 20 *μ*m channel; the number ratio of *Pf*-IRBC to HRBC is 1 : 9.

In Figure 13, trajectories of cell center are plotted for an arbitrary time period of 20 ms for the 10 *μ*m and 20 *μ*m channels with 45%-*H*_ct_. By taking into account the RBC's size, cell free layer in the 10 *μ*m channel is about 1.5 *μ*m. Cell free layer in this 20 *μ*m channel is estimated as 1.9 *μ*m which is 1 *μ*m less than the averaged distance between the outmost cell center and the adjacent wall. Note that in Figure 13(c) the cell free layers at the top and the bottom wall seem to be slightly different. The reason for this difference is that the *Pf*-IRBCs are located close to the bottom wall, which causes the increase of the cell free layer. Therefore, the cell free layer thickness in Figure 13(c) is the averaged value at the top and the bottom wall. The results in the current study are bigger than the cell free layer thickness found by Maeda et al. [37] who reported cell free layers in the scope of 1–1.8 *μ*m for blood vessel diameters 10–40 *μ*m with *H*_ct_ = 45%. The slight increase of the CFL in the 20 *μ*m channel may be caused by the elevation of the local hematocrit: the RBC region is denser than normal thus generating a thicker CFL near the wall.

**Figure 14:**
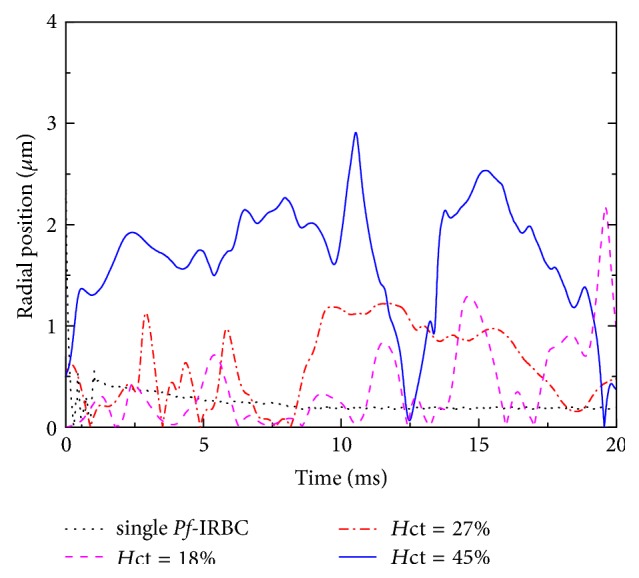
Time history of the radial location of the *Pf*-IRBC in the 10 *μ*m channel for various simulation conditions. The radial location is defined as the distance between the center of the *Pf*-IRBC and the vessel center.

**Figure 15:**
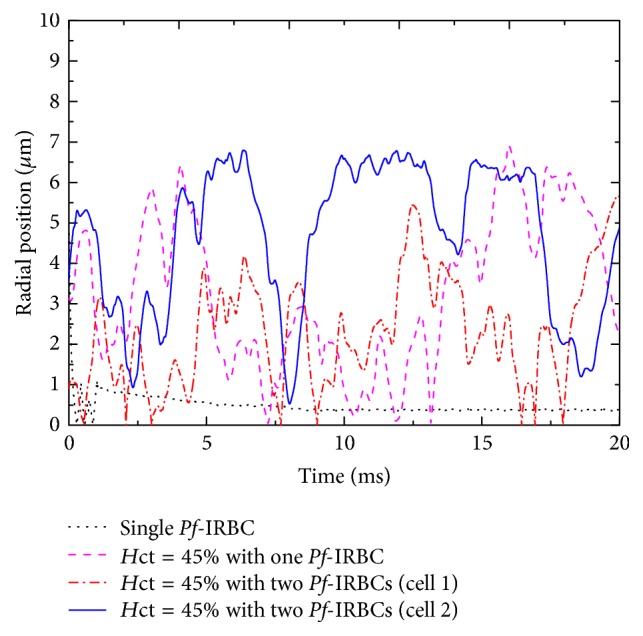
Time history of the radial location of the *Pf*-IRBC in the 20 *μ*m channel for various simulation conditions. The radial location is defined as the distance between the center of the *Pf*-IRBC and the vessel center.

Figures 14 and 15 show the trajectory of the radial location of the cell center of the *Pf*-IRBC in the 10 *μ*m and 20 *μ*m channels, respectively. The figures are illustrated for an arbitrary time duration of 20 ms for the non-single-cell cases. In the case of a single *Pf*-IRBC without HRBCs, it shifts to the center of the vessel gradually even when it is in the beginning settled close to the wall. The equilibrium position is not exactly at the centerline of the vessel due to its small degree of deformability.

Significant margination of *Pf*-IRBC is observed in the case of *H*_ct_ = 45%, however not for the case of *H*_ct_ = 18% and 27% in Figure 14. In the two latter cases, the *Pf*-IRBC can occasionally move toward the vessel wall because of the interaction of the next HRBC. However, this interaction did not last long because of the relatively low *H*_ct_ value. Until the following HRBCs once again catch up to the *Pf*-IRBC, the *Pf*-IRBC moves almost along the channel center. Figure 7 displays the representative visualization of the distribution of RBCs for *H*_ct_ = 45% in the 10 *μ*m channel. The HRBCs can flow parallel to the axis of the channel and thus can pass the *Pf*-IRBC and push the *Pf*-IRBC away from the center. The *Pf*-IRBC can thus migrate to the vessel wall and flow close to the endothelial cells. When the surrounding HRBCs leave the *Pf*-IRBC, they return to the center of the channel promptly. On some occasions, the *Pf*-IRBC shifts back to the center of the channel. In this situation, the process will restart and the *Pf*-IRBC migrates to the wall once again.

Similar margination of *Pf*-IRBCs has been observed in the 20 *μ*m channel (Figure 15). When there is only one *Pf*-IRBC and *H*_ct_ is 45%, the *Pf*-IRBCs move toward the vessel wall and can reach the CFL occasionally. In the two *Pf*-IRBCs' case, it seems that the margination effect for one *Pf*-IRBC is more obvious than the other *Pf*-IRBC in the time period illustrated. As shown in Figure 9(b), HRBCs sometimes slip into the opening between channel wall and one *Pf*-IRBC and then push the *Pf*-IRBC away from the wall in this high-*H*_ct_ condition. The *Pf*-IRBC may not have a chance to reach the CFL for a long time.

## 5. Discussion

Our simulation demonstrates that the *Pf*-IRBCs move slower than HRBCs and cause local *H*_ct_ increase around the *Pf*-IRBC. The former effect is qualitatively in agreement with numerical results in [38]. The latter effect is severe in high-*H*_ct_ conditions. In the 10 *μ*m channel with 18%  *H*_ct_ (Figure 3), the volume fraction is sometimes elevated. However, this local increase of *H*_ct_ cannot be maintained for long and the overall cell distribution is almost uniform. Although we can see the flow disturbance through the pressure field in this case, obviously this disturbance is not enough to maintain high-*H*_ct_ region. On the other hand, in the same channel as *H*_ct_ increases to 27% (Figure 5), the HRBCs and *Pf*-IRBC develop a train of cells. The *Pf*-IRBC and the following HRBC are close enough to have contact with each other and the HBCs are accumulated in the downstream region where the local *H*_ct_ is elevated to around 40% (Figure 11(a)). In the 45%  *H*_ct_ condition (Figure 7), the train configuration is not demonstrated and the volume fraction is sometimes raised to about 50% or even 60% in the *Pf*-IRBC region. The volume fraction, however, immediately falls down since, in the upstream zone, the volume fraction is low, whereas, in the vessel of 20 *μ*m, no significant local *H*_ct_ increase has been seen in the simulations (Figure 9). Similar trends have been found for the discharge hematocrit values as well (Figure 12). However, we observe an increase of cell free layer thickness which means the RBCs are more tightly packed in the center region. This result is qualitatively consistent with* in vitro* findings [39]. Therefore, the slower moving of *Pf*-IRBC and its ability to block the HRBCs are the mechanism of local fluctuation of *H*_ct_. That is, a relatively high concentration of RBCs and narrow tube is necessary, to keep the contact of the HRBCs with the *Pf*-IRBC. Note that the present simulations do not incorporate the effects of adhesion interactions, which will give rise to a more severe and longer duration of aggregation of RBCs. Moreover, the pattern of WSS variation suggests that it could be the direct result of *H*_ct_ and CFL variations.

Generally, HRBCs experience lateral migration because of their deformability and tend to accumulate at the center of the blood vessel therefore leaving a cell free layer close to the vessel wall (Figure 13). When the HRBCs are invaded by the* Plasmodium falciparum* parasite, they have been known to become less deformable. However, they still retain some deformability which makes them migrate to the centerline of the blood vessel in the single *Pf*-IRBC case (Figures 14 and 15). Margination of *Pf*-IRBC that occurred in our simulations especially in the high-*H*_ct_ conditions suggests that the hydrodynamic interaction between the *Pf*-IRBC and HRBCs plays a key role. Although our simulations were conducted in 2D conditions, the results are qualitatively in agreement with experimental data by Hou et al. [15]. The quantitative discrepancy may be attributed to unlikely experimental circumstances such as the size of channel and *H*_ct_ values. In [15], a 10 *μ*m by 15 *μ*m rectangular channel was used. They observed significant margination of *Pf*-IRBCs for blood sample with *H*_ct_ = 40%. However, the margination effect was insignificant for blood sample of *H*_ct_ = 10%. We have obtained the similar result that the RBCs in 18%-*H*_ct_ blood show nearly uniform distributions. We illustrate that the *Pf*-IRBC can migrate and be away from the vessel wall in the 27%-*H*_ct_ condition in the 10 *μ*m channel. However, it migrates back to the channel center because of its deformability. This process of moving away and back to the center could happen repeatedly if the simulation time is sufficiently long. Moreover, they reported that some *Pf*-IRBCs still flowed close to the center in the high-*H*_ct_ samples. We have observed a similar phenomenon in our simulations as shown in Figure 15. This is attributed to the continuous interaction between HRBCs and the *Pf*-IRBC because the *Pf*-IRBC is always surrounded by HRBCs. The HRBCs deform more and move faster than the *Pf*-IRBC. The HRBCs can slide into the gap between the *Pf*-IRBC and vessel wall and then push the *Pf*-IRBC away from the wall in high-*H*_ct_ blood (Figure 9(b)). Thus, the *Pf*-IRBCs cannot be separated completely from HRBCs even in long enough channels. This result will help in designing and fabricating microfluidic devices.

Although we ignore the cytoadhesion of the *Pf*-IRBC to the endothelial cells in this study, this property has been reported experimentally and is believed to be a factor that enhances margination of *Pf*-IRBCs [40]. In their study, the effect of *H*_ct_ on the adhesion and rolling to human dermal microvascular endothelial cells has been investigated* in vitro*. It has been found that both adhesion and rolling were intensified with raising *H*_ct_ from 10% to 20%. They predicted that this enhancement was due to the rise in cell margination and contact period with endothelial cells. The results of *H*_ct_-dependent margination in the present study give support to their prediction. *Pf*-IRBCs will migrate to the vessel wall under a long contact period with HRBCs in high-*H*_ct_ conditions in the 10 *μ*m and the 20 *μ*m vessels, whereas the margination is not obvious because of the occasional contacts in 18%-*H*_ct_ sample.

## 6. Conclusions

This study illustrates the effects of *Pf*-IRBC on microscopic blood flow owing to hydrodynamic interaction between the HRBCs and *Pf*-IRBC. In the 10 *μ*m vessel, HRBCs and a *Pf*-IRBC form a train of cells. Even in low-*H*_ct_ conditions, HRBCs and a *Pf*-IRBC form a temporary train because the low volume concentration of RBCs does not submit continuous interaction. The local hematocrit is elevated to even above 50% around the *Pf*-IRBC in high-*H*_ct_ conditions while *H*_ct_ in other areas is deceased, resulting in local *H*_ct_ fluctuation. Note that the cytoadhesion is not considered in this study; thus this aggregation of RBCs is mainly caused by the slow moving of *Pf*-IRBCs. The variation of WSS can be related to the fluctuation of *H*_ct_ close to the wall and the distance between the wall and the nearest RBC. It is also observed that the *Pf*-IRBC tends to migrate to the vessel wall in high-*H*_ct_ condition because of the continuous interaction between the *Pf*-IRBC and the HRBCs. The *Pf*-IRBC may migrate to the vessel wall and it can also move back to the channel center. We conclude that our results provide some insight into the hemodynamic influences of *Pf*-IRBCs in microvascular system and will help in improving microfluidic devices.

## 7. Limitations and Future Research

Firstly, we did not consider adhesive property of *Pf*-IRBCs, which is due to the formation of nanoscale knobs that protrude from the cell membrane within 24–48 hours of infection. Numerical study [38] on *Pf*-IRBCs with adhesive property has shown interesting phenomena including rolling motion on endothelial cells and complex interaction with HRBCs. It is reasonable to assume that when the cytoadherence of infected RBCs is included, the hemodynamics of blood flow will be significantly altered and the cytoadherence of *Pf*-IRBCs to endothelial cells will be incorporated in the future investigation.

Secondly, we described *Pf*-IRBCs as a hardened RBC characterized by a stiffer membrane which is a simple assumption. Imai et al. [25] modeled malaria parasite as a rigid core inside the *Pf*-IRBC. Zhang et al. [41] hypothesized that the nanoscale knobs that cause the stickiness of the cell membrane also cause the membrane to stiffen. Therefore modeling the cell membrane with multiple nanoscale knobs with mechanical property different from the elastic membrane will be a direction of our future research.

Finally, the current study used two-dimensional simulation scheme in a rectangular microchannel, which is a significant limitation in terms of direct interpretation of the computational results and comparison with clinic data because there is some discrepancy between our simulations and real-world system. For instance, the hematocrit and the flow rate are the major parameters that will be discrepant from the real values. For this reason, we only make qualitative comparison with experimental results in the paper. Realistic three-dimensional simulations with physiologically relevant hematocrit contents are needed in the future study to obtain more accurate results and to quantitatively compare with experimental data.

## Figures and Tables

**Table 1:** Parameters used for the simulations.

Parameter	Symbol	Value
Blood plasma density	*ρ*	1.0 g/cm^3^
Blood plasma viscosity	*μ*	1.2 cp
Axial pressure gradient in 10 *μ*m channel	*P*_*x*_	600 KPa/m
Axial pressure gradient in 20 *μ*m channel	*P*_*x*_	400 KPa/m
Radius of the circle in RBC model	*r*_0_	2.8 *μ*m
Number of springs in RBC model	*N*	76
Membrane mass in RBC model	*m*	2.0 × 10^−4^ g
Membrane viscosity in RBC model	*γ*	8.8 × 10^−7^ N·s/m
Spring constants for HRBC membrane	*k*_*l*_ & *k*_*b*_	1.0 × 10^−12^ Nm
Spring constants for *Pf*-IRBC membrane	*k*_*l*_ & *k*_*b*_	2.0 × 10^−11^ Nm
Width of the channel	*L*	30 *μ*m
Height of the channel	*D*	10 *μ*m, 20 *μ*m
Mesh size	*h*	1/80 *μ*m
Time step	Δ*t*	1 × 10^−5^ ms
